# Rose Prickles and *Asparagus* Spines – Different Hook Structures as Attachment Devices in Climbing Plants

**DOI:** 10.1371/journal.pone.0143850

**Published:** 2015-12-02

**Authors:** Friederike Gallenmüller, Amélie Feus, Kathrin Fiedler, Thomas Speck

**Affiliations:** Plant Biomechanics Group Freiburg, Faculty for Biology, Botanic Garden of the Albert-Ludwigs-University, Freiburg, Germany; McGill University, CANADA

## Abstract

Functional morphology and biomechanical properties of hook structures functioning as attachment devices in the leaning climbers *Rosa arvensis*, *Rosa arvensis* ‘Splendens‘, *Asparagus falcatus* and *Asparagus setaceus* are analysed in order to investigate the variability in closely related species as well as convergent developments of hook structure and properties in distant systematic lineages (monocots and dicots). Prickles and spines were characterised by their size, orientation and the maximum force measured at failure in mechanical tests performed with traction forces applied at different angles. In *Rosa arvensis* and *Rosa arvensis* ‘Splendens‘ three types of prickles differing largely in geometrical and mechanical properties are identified (prickles of the wild species and two types of prickles in the cultivar). In prickles of *Rosa arvensis* no particular orientation of the prickle tip is found whereas in the cultivar *Rosa arvensis* ‘Splendens‘ prickles gradually gain a downward-orientation due to differential growth in the first weeks of their development. Differences in mechanical properties and modes of failure are correlated to geometrical parameters. In *Asparagus falcatus* and *Asparagus setaceus* spines are composed of leaf tissue, stem tissue and tissue of the axillary bud. Between species spines differ in size, orientation, distribution along the stem, tissue contributions and mechanical properties. The prickles of *Rosa arvensis* and its cultivar and the spines of the studied *Asparagus* species have several traits in common: (1) a gradual change of cell size and cell wall thickness, with larger cells in the centre and smaller thick-walled cells at the periphery of the hooks, (2) occurrence of a diversity of shape and geometry within one individual, (3) failure of single hooks when submitted to moderate mechanical stresses (F_max_/basal area < 35 N/mm²) and (4) failure of the hooks without severe stem damage (at least in the tested wild species).

## Introduction

Leaning plants are a particular group of climbers that do not possess specialised climbing organs such as twining stems, tendrils or adhesive roots and establish only a loose attachment to their supporting structures. They are also referred to as “semi-selfsupporting plants”[1, 2]. Some of these species develop hooks which prevent the slipping off of the axes from the supporting structures and can also be characterised as “hook climbers”[2–4]. Here we analyse the functional morphology and mechanical properties of two largely different types of hooks: prickles (derived from the epidermis) and spines (derived from leaves). In order to determine (1) convergent developments in hook formation in leaning climbers from different systematic groups, and (2) the variability of hook structure and properties in closely related species we investigated prickles of the dicotyledonous *Rosa arvensis* and the cultivar *Rosa arvensis* ‘Splendens‘ as well as spines of the monocotyledonous *Asparagus falcatus* and *Asparagus setaceus*. The woody *Rosa* species and the cultivar as well as the two herbaceous *Asparagus* species are typical hook climbers and develop stems that become mechanically instable after having reached a certain length and then „lean on”supporting structures in their vicinity. The hooks (prickles in *Rosa arvensis* and the cultivar and spines in the *Asparagus* species) help to secure the attachment to these supporting structures, although it can be assumed that they simultaneously function as defence against herbivores. Rose prickles are typically derived from the epidermis and the tissue just below, and have been described in a large variety of forms in different rose species [5]. The spines of different *Asparagus* species have been described as outgrowths from the scale-like leaves covering the long axes by [6, 7].

## Materials and Methods

Experimental plants were cultivated in the open field (*Rosa arvensis* and *Rosa arvensis* ‘Splendens‘) or in pots in a greenhouse (*Asparagus setaceus* and *Asparagus falcatus*) of the Botanical Garden of the University of Freiburg. Since considerable morphological differences between short and long axes have been described for other rose species [8, 9], the morphology of stems was studied in all test plants prior to the selection of stems and hooks for the mechanical and anatomical investigations. All experimental plants were provided with trellises. All tests were performed with fresh material within 2 hours after cutting the stem segments. Stem segments were stored in wet tissues prior to the tests and kept humid in a vapour-filled chamber during the drying of the glue (approximately 15 minutes) in order to prevent desiccation. A test series on *Rosa* prickles after storage periods from 20 to 120 minutes revealed no influence of the storage time on the obtained values of maximum force at failure (*Fmax/basal area*).

**Fig 1:**
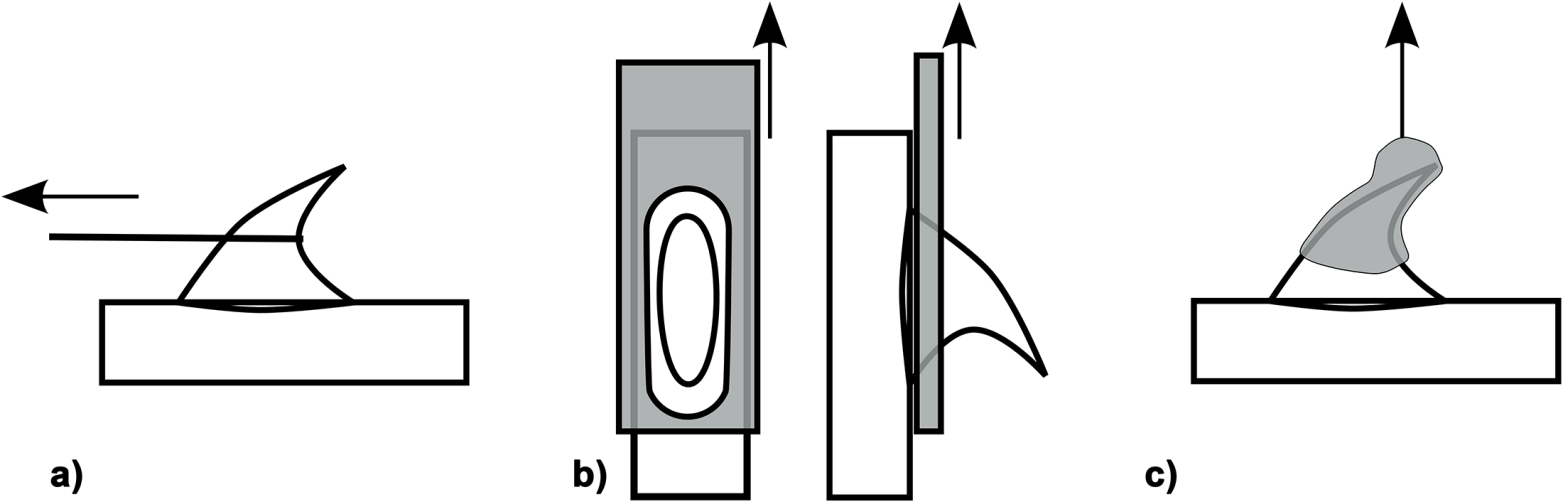
Types of mechanical tests. Mechanical tests were performed with a) traction applied via a Kevlar loop resulting in a combination of bending and shear stresses (*Rosa* prickles and *Asparagus* spines), b) traction parallel to the stem applied at the base of the hook via perforated aluminium plates (*Rosa arvensis* ‘Splendens‘ type II prickles, resulting in pure shear stresses) and c) traction perpendicular to the stem and applied to the whole *Rosa* prickle, resulting in pure tension stress.

**Fig 2:**
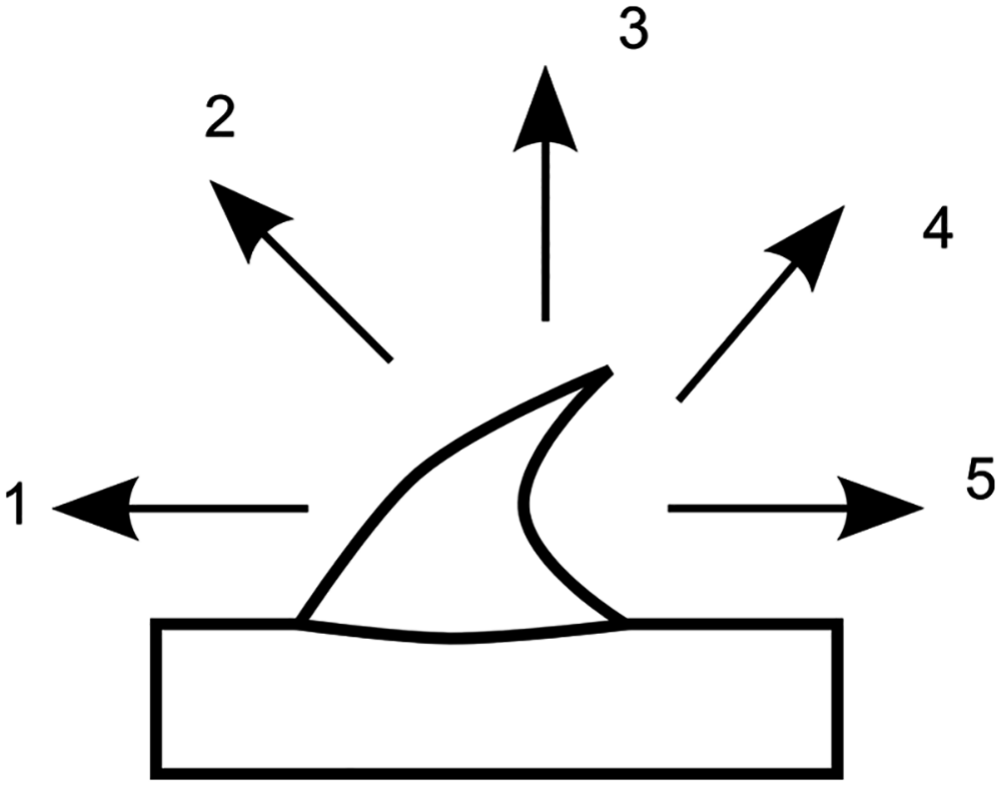
Directions of traction forces in the mechanical tests. Traction force was applied with a Kevlar loop. 1: parallel to the axis and oriented to the apex, 2 and 4: with an angle of +/-45°, 3: with an angle of 90°, 5: parallel to the axis and oriented to the stem base.

Mechanical tests of prickles and spines were performed using a tensile testing device consisting of a linear table, a lifting table, a 50N force transducer (31E, Honeywell, Columbo OH, USA) and fixing devices for samples adjustable in angles from 0–45° to the direction of the tension force. Force/displacement data were recorded with LabVIEW 8.2.1 (National Instruments, Austin, Texas, USA). Stem segments were cut near the hook or spine chosen for testing and glued to aluminium plates fitting in the fixing devices, using superglue (Uhu Sekundenkleber flüssig) on the stem segment. In order to increase adhesion the aluminium plates were pre-treated with a two-component adhesive (Pattex Stabilit Express) 24 hours before the experiments. Hooks were pulled via a Kevlar loop (JENZI TMX-Vorfach, Plüderhausen, Germany) composed of a several parallel Kevlar fibres (*Asparagus* spines) or a plaited bundle of Kevlar fibres (*Rosa* prickles) at different angles between the longitudinal axis of a stem segment and the direction of the tension force (Figs 1a and 2) [10]. As Kevlar loops positioned at the tip of the hooks slipped from the tip to the vertex point when the pulling force was applied the Kevlar loops were directly positioned at the vertex point at the beginning of each test. In order to obtain angles of 90° T-shaped aluminium plates were used. *Rosa arvensis* ‘Splendens‘ type II prickles were additionally tested (1) by fixing the whole hook structure between clamps and pulling it perpendicularly to the stem, and (2) by generating shear stress with a traction force parallel to the stem and applied at the base of the hook (Fig 1b and 1c). In the latter case hook structures were fitted through perforations in aluminium plates mounted in the fixing devices and experiments were performed with an Hegewald & Peschke testing machine using a 100N force transducer (Instron, Static Load Cell, High Wycombe, USA) (Fig 1b).

All statistical tests were performed using R (version 2.12.0, libraries ‘car’ and ‘dunn’, parametric data, two groups, equal variances: Student’s t-test, unequal variances: Welch’s t-test, non parametric data, 2 groups: Mann-Whitney U-Test, > 2 groups: Kruskal-Wallis and Tukey’s-Test).

**Fig 3:**
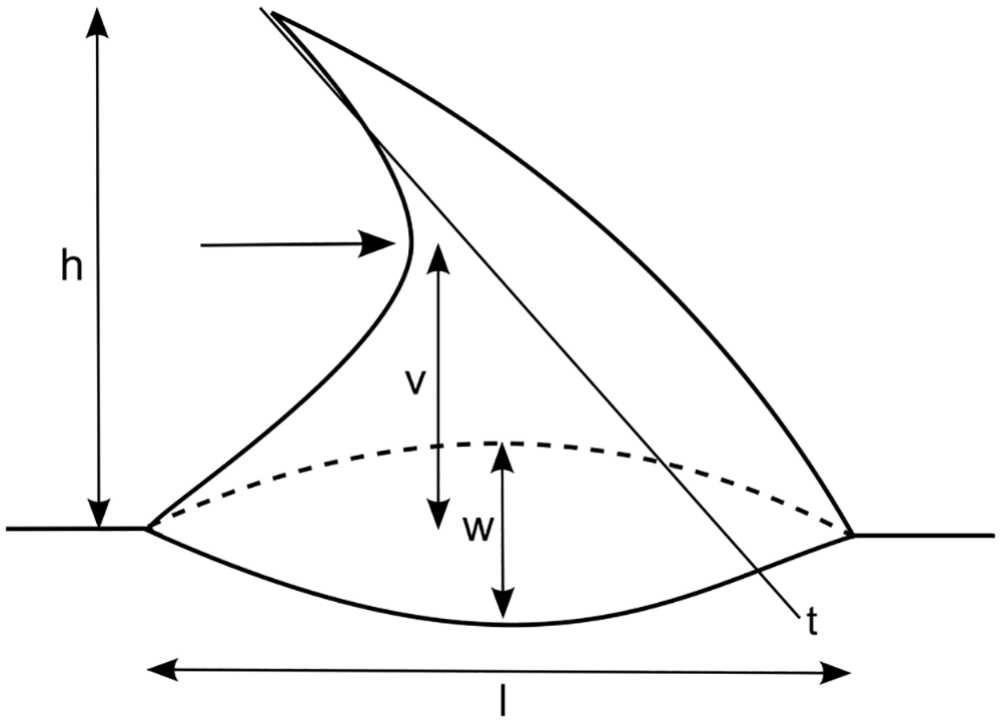
Analysis of prickle and spine geometry. Measured parameters are h (height), l (length of prickle base), w (maximum width of prickle base), v (distance of vertex from prickle base) and the angle between t (inner tangent) and the stem.

Geometry of the tested prickles and spines was characterised determining the height, basal length, basal width and, if applicable, the vertex position of the hook as well as the angle formed with the longitudinal axis of the stem (Fig 3). Tested segments were stored in FAA fixative and cut with a freezing microtome. Cuts of *Rosa* prickles were stained with FCA. In cuts of *Asparagus* spines a better contrast between different tissues was obtained with toluidine blue.

## Results

**Fig 4:**
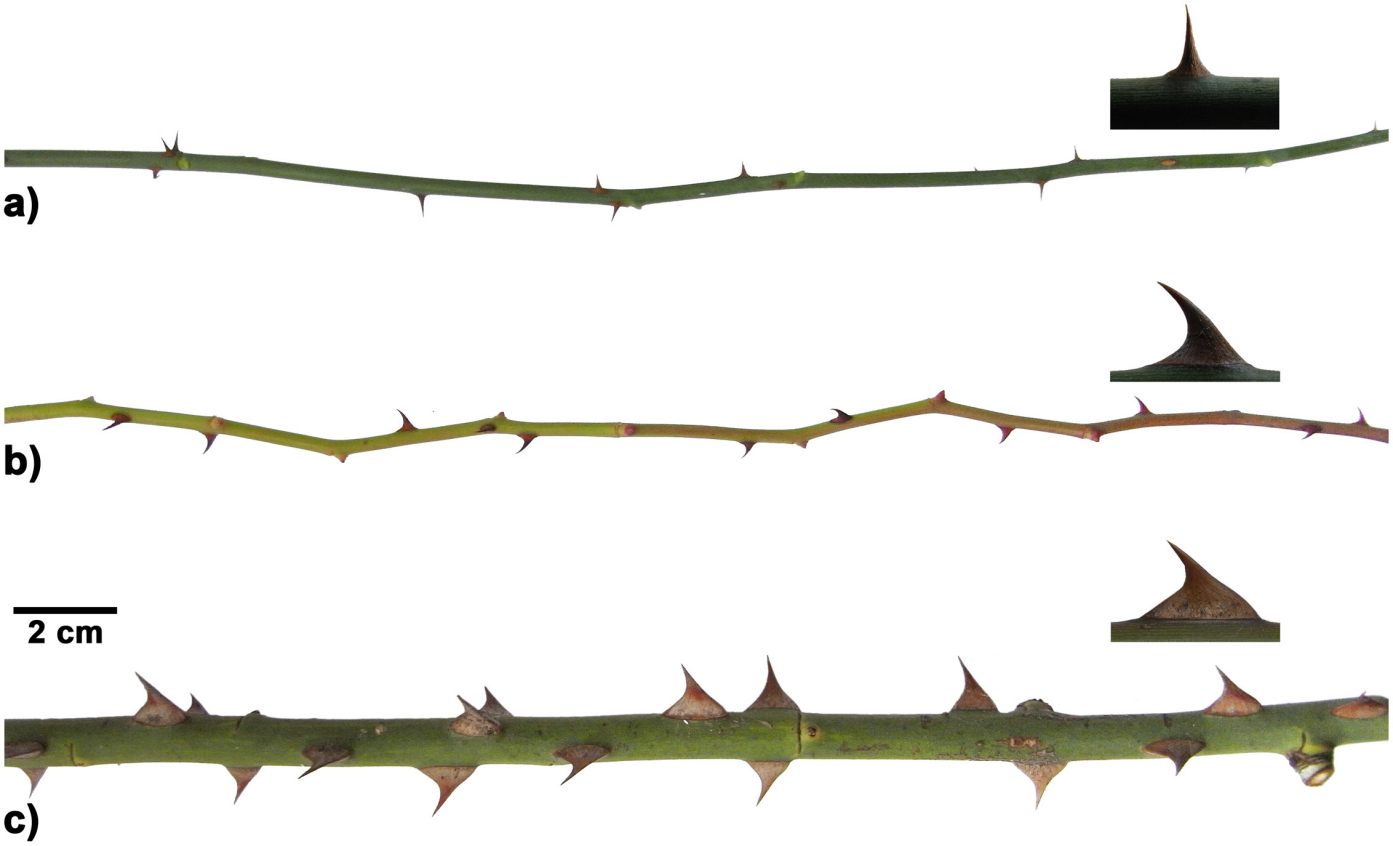
Morphology of different types of long axes and prickles in *Rosa arvensis* and *Rosa arvensis* ‘Splendens‘. a) long axis of *Rosa arvensis*, b) long axis of *Rosa arvensis* ‘Splendens‘, Type I, c) long axis of *Rosa arvensis* ‘Splendens‘, Type II.

**Fig 5:**
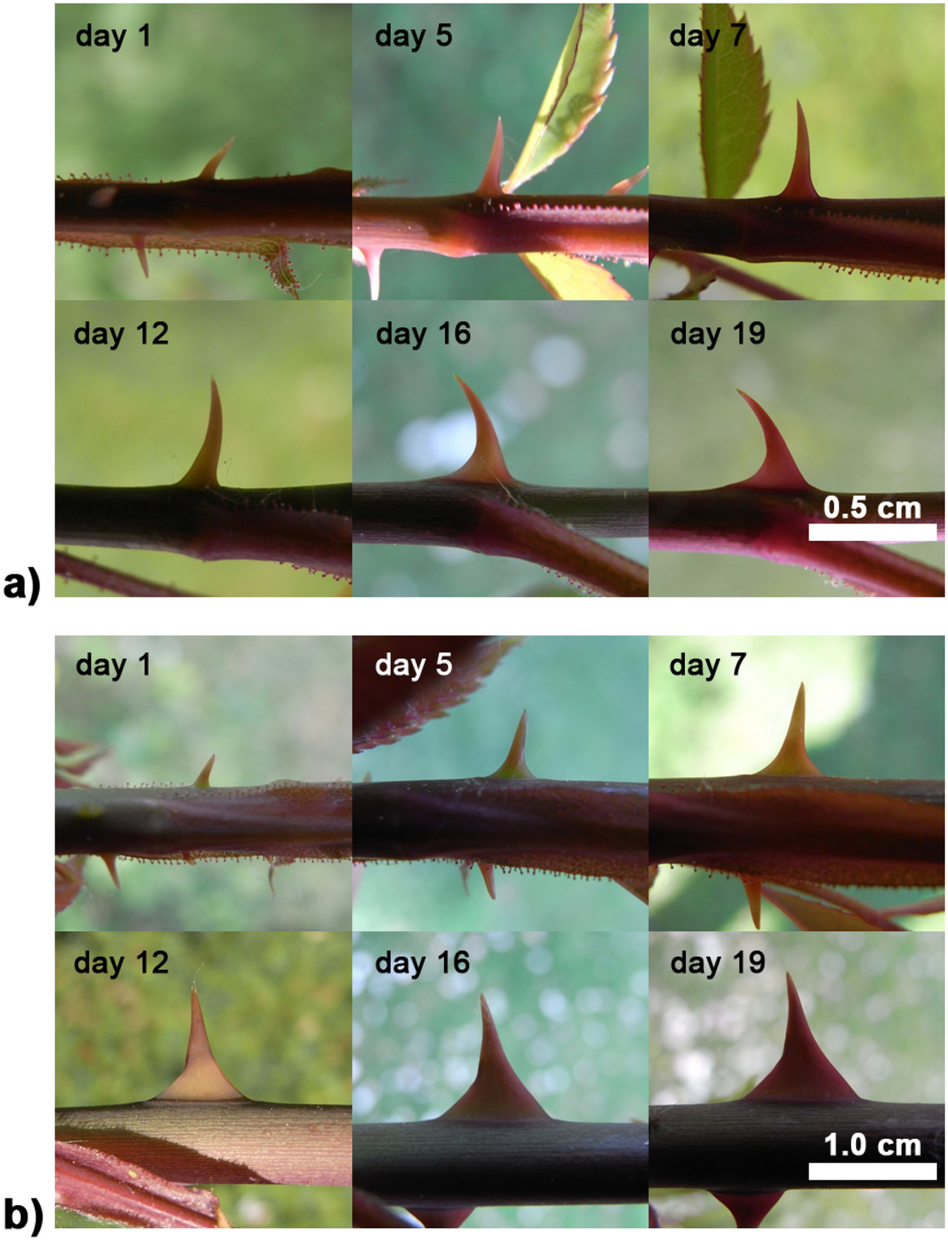
Development of prickles in the different types of long axes of *Rosa arvensis* ‘Splendens‘. a) type I, b) type II. In the younger stages (day 1–7) the glandular margins of adjacent leaf petioles lie flat against the axis (stem apex to the right).

In both *Rosa arvensis* and *Rosa arvensis* ‘Splendens‘ a clear distinction between short axes and long axes was possible. In addition, two types of long axes differing in their morphology were identified in the cultivar *R*. *arvensis* ‘Splendens‘ and named long axis type I and type II. Comparing these two types of long axes in the cultivar *R*. *arvensis* ‘Splendens‘ reveals differences in geometry and mean diameter of the axes as well as differences in geometry, size and orientation of the prickles, with significantly larger axes (with a higher diameter) and significantly larger prickles in type II (Table 1, Fig 4). In fully developed prickles of *Rosa arvensis* no particular orientation of the prickle tip towards base or apex of the stem is found and the prickles show a median angle between the axis and inner prickle tangent of 91.8°. On the contrary, in both types of long axes of the cultivar *Rosa arvensis* ‘Splendens‘ prickles are clearly oriented downwards (towards the base of the stem) with a median angle between the stem and the prickle tangent of 47.1° in type I and of 53.5° in type II (Table 1, Fig 4). However, this holds only for fully developed prickles. In early stages of development the prickles even display an upward-orientation before they gradually gain their downward-orientation in adult stages due to differential growth (Fig 5). No variation of prickle size and/or orientation was observed between different segments of the respective stems types.

**Table 1:** Morphology of long axes and geometry of the tested rose prickles.

	long axes of *R*. *arvensis*	long axes of *R*. *arvensis* ‘Splendens‘, type I	long axes of *R*. *arvensis* ‘Splendens‘, type II
axis geometry	metamers build a straight axis	formation of angles between metamers	metamers build a straight axis
mean axis diameter [mm]	2.7 (2.4, 3.0)	2.8 (2.4, 3.9)	5.9 (4.7, 6.3)
mean height of prickles [mm]	3.3 (2.7, 3.9)	4.8 (4.4, 5.4)	7.5 (6.2, 8.4)
mean length of prickle base (mm)	2.0 (1.8, 2.3)	5.9 (5.0, 7.1)	10.9 (9.4, 13.3)
mean width of prickle base	1.3 (1.2, 1.5)	1.9 (1.6, 2.1)	2.8 (2.5, 3.4)
vertex position (distance from prickle base) [mm]	non identifiable	2.0 (1.7, 2.3)	3.8 (3.2, 4.6)
prickle orientation [°]	91.8 (83.0, 101.0)	47.1 (41.4, 54.1)	53.5 (50.1, 59.3)

The specified values are medians and lower and upper quartiles (in brackets). Number of samples are n = 36 (*Rosa arvensis*), n = 141 (*Rosa arvensis* ‘Splendens‘ type I) and n = 149 (*Rosa arvensis* ‘Splendens‘ type II). All differences are significant (Kruskal-Wallis test and Dunn’s test, p < 0.001) with exception of the mean axis diameter compared between long axes of *Rosa arvensis* and *R*. *arvensis* ‘Splendens‘ type I, where no significant differences are found.

**Fig 6:**
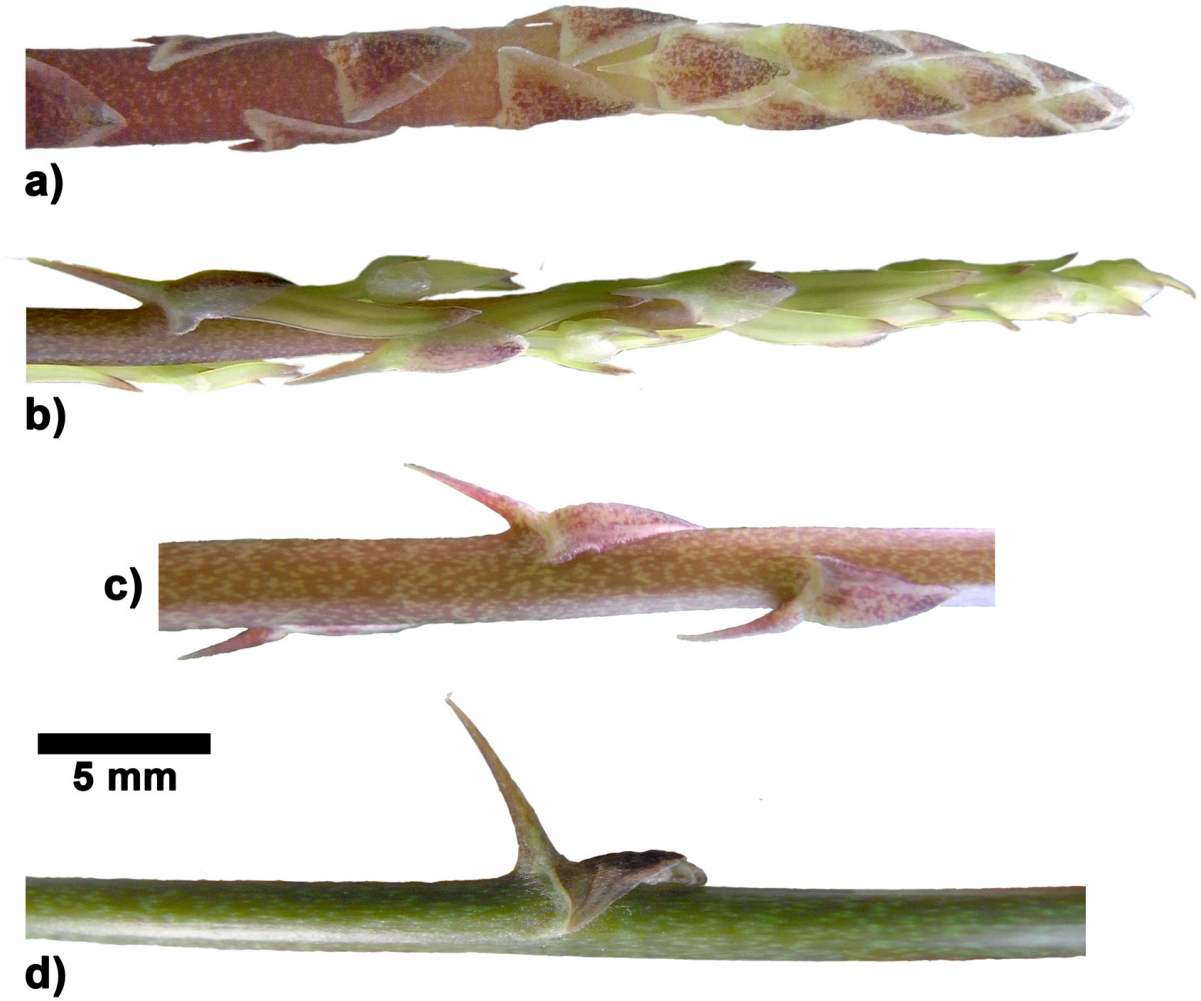
Morphology of stems and spines in *Asparagus falcatus*. a) shoot tip with spines lying flat against the axis, b) same shoot tip 5 days later with emerging axillary buds, c) transition zone with spines elevated at different angles, d) zone near the apex with fully developed spines erected at higher angles (≤ 80°).

**Fig 7:**
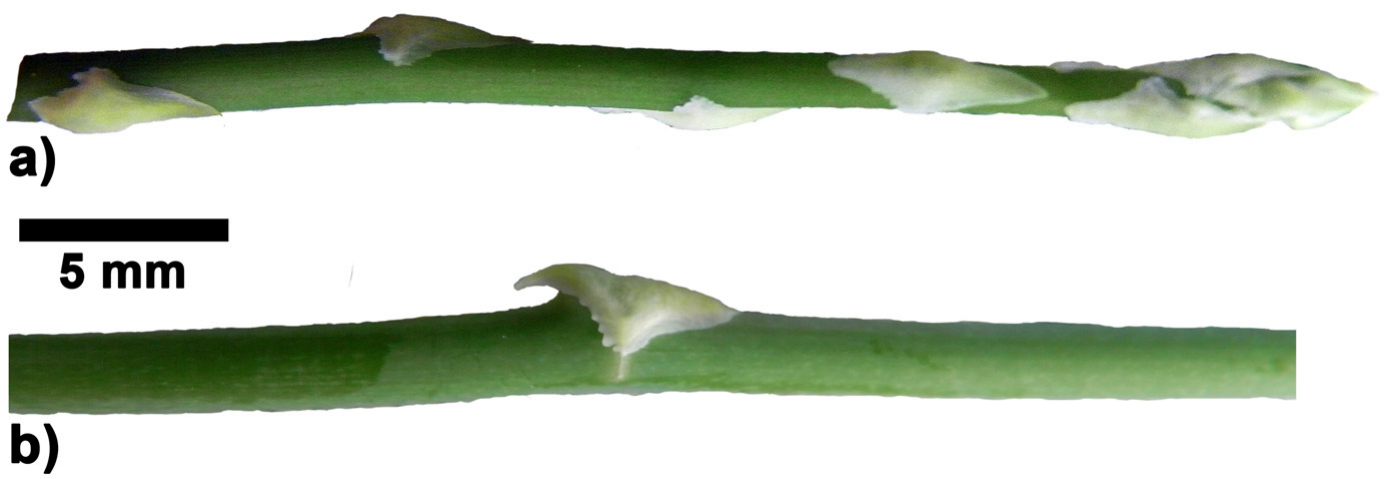
Morphology of axes and spines in *Asparagus setaceus*. a) shoot tip with young, not yet fully developed spines, b) middle segment with fully differentiated spine.

On the contrary, in both *Asparagus setaceus* and *Asparagus falcatus* the morphology of the spines varies with their position on the stems. In the basal segments spines do not become fully developed and remain small and soft structures. In the middle and apical stem segments of both species the scale-like leaves develop into spines whose size gradually increases towards the apex (with exception of the newly and not fully developed smaller young spines a few centimetres underneath the apex, Figs 6 and 7). In the studied individuals of both species the length of the largest spines amounted to approximately 5 mm. However, the spines of *A*. *setaceus* and *A*. *falcatus* differ considerably in shape.

In *Asparagus falcatus* the small spines of basal stem segments lie flat against the axis. In middle and apical stem segments increasingly protruding spines are produced (Fig 6). The final angle formed by fully developed spines eventually depends on the position of the spines on the stems, with largest angles in apical segments. The most erected spines form an angle of 80° with the axis (Fig 6.). Unlike the observations made for the rose prickles which change their shape and orientation during ontogeny the overall shape of the *A*. *falcatus* spines (varying along the stems) is fixed as soon as the spines emerge (Fig 6). This means that the apical meristem undergoes different phases, producing small flat spines in early stages and larger spines with increasing angles to the longitudinal axis of the stem in later stages (Fig 6). On the contrary, in *Asparagus setaceus* all spines found along the middle and apical stem segments show a similar shape and are all slightly bent towards the stem. Like spines in *A*. *falcatus* spines in *A*. *setaceus* obtain their overall shape already visible in the first developmental stages (Fig 7). Spines in *Asparagus falcatus* differ significantly in height, length and width from spines in *Asparagus setaceus* (Table 2).

**Table 2:** Morphology of long axes and geometry of prickles in *Asparagus falcatus* and *Asparagus setaceus*.

	*A*. *setaceus*	*A*. *falcatus*
axis diameter [mm]	2.5 (2.4, 2.7)	2.1 (2.0, 2.4)
height of spines [mm]	1.6 (1.4, 1.8)	3.1 (2.8, 3.9)
length of spine base [mm]	5.9 (5.3, 6.3)	4.5 (3.4, 4.7)
width of spine base [mm]	2.3 (2.1, 2.5)	1.5 (1.1, 1.8)
spine orientation [°]	33.2 (27.3, 38.9)	55.4 (46.5, 60.5)

The specified values are medians and lower and upper quartiles (in brackets). All differences are significant (Mann-Whitney U-Test and Welch’s t-test, p < 0.001). Number of samples are n = 36 (*Asparagus falcatus*) and n = 27 (*Asparagus setaceus*).

**Fig 8:**
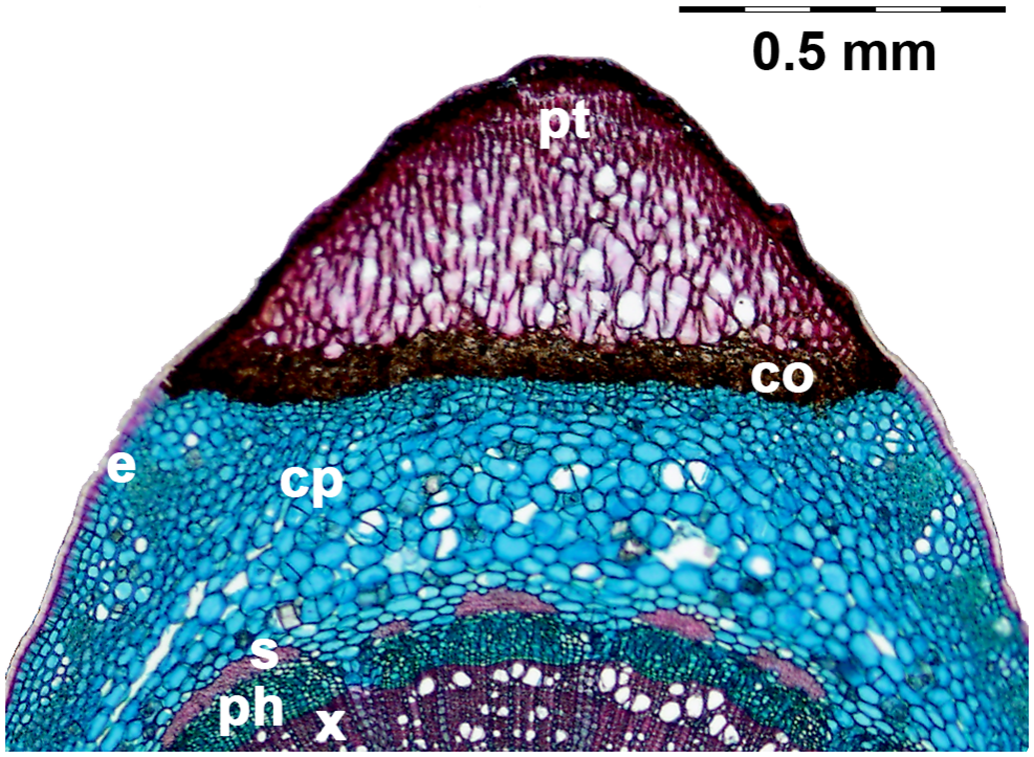
Transverse section of a *Rosa arvensis* ‘Splendens‘ stem with prickle (type I). The section is stained with FCA. x: xylem, ph: phloem, s: sclerenchyma, cp: cortical parenchyma, e: epidermis, co: cork, pt: lignified prickle tissue.

All investigated prickles of *Rosa arvensis* and *Rosa arvensis* ‘Splendens‘ (type I and type II) show a similar anatomical structure with a basal cork layer between the cortical parenchyma of the stem and the prickle tissue (Fig 8). The lignified prickle tissue displays a gradual variation of cell size with largest cells in the centre and smaller, thick-walled cells at the periphery of the prickles. This is conform to the findings of [11], who describe fully differentiated prickles of different Rosoideae (several raspberry cultivars and redberry cultivars as well as *Rosa hybrida* ‘Radtko’) as completely lignified structures and opposed to a younger, still growing stage with only partial lignification.

**Fig 9:**
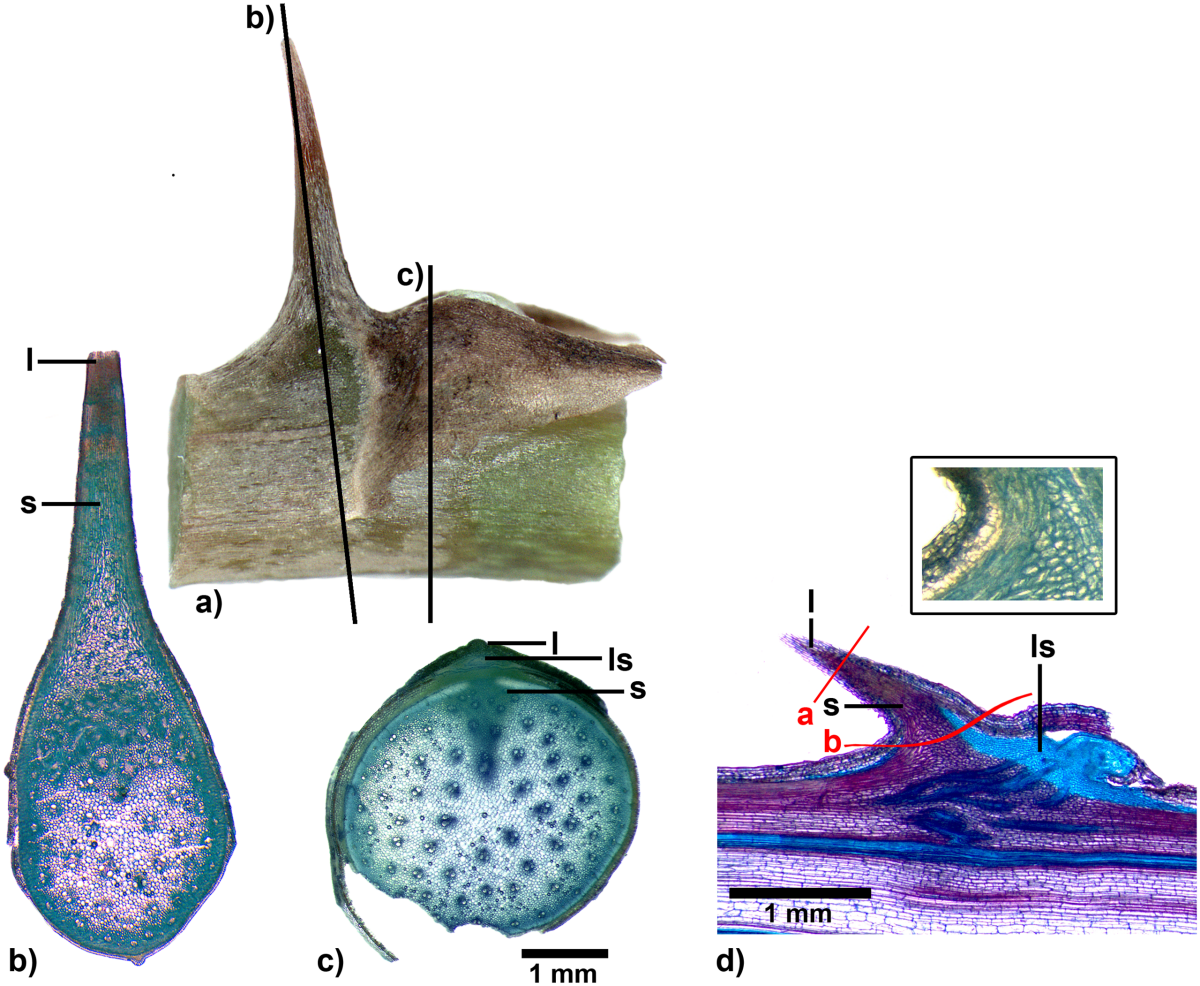
Spine of *Asparagus falcatus*. a) Side-view of a spine with split leaf structure and emerging axillary bud, black lines indicate section planes of cross sections b) and c), b) and c) cross sections at different planes (from different specimens) both stained with toluidine blue (box: close up from a different, unstained cut), d) longitudinal section stained with toluidine, red lines indicate modes of failure observed in the mechanical tests, a: failure of the tip, b: failure of the whole spine). l: leaf tissue, s: stem tissue, ls: lateral shoot tissue.

**Fig 10:**
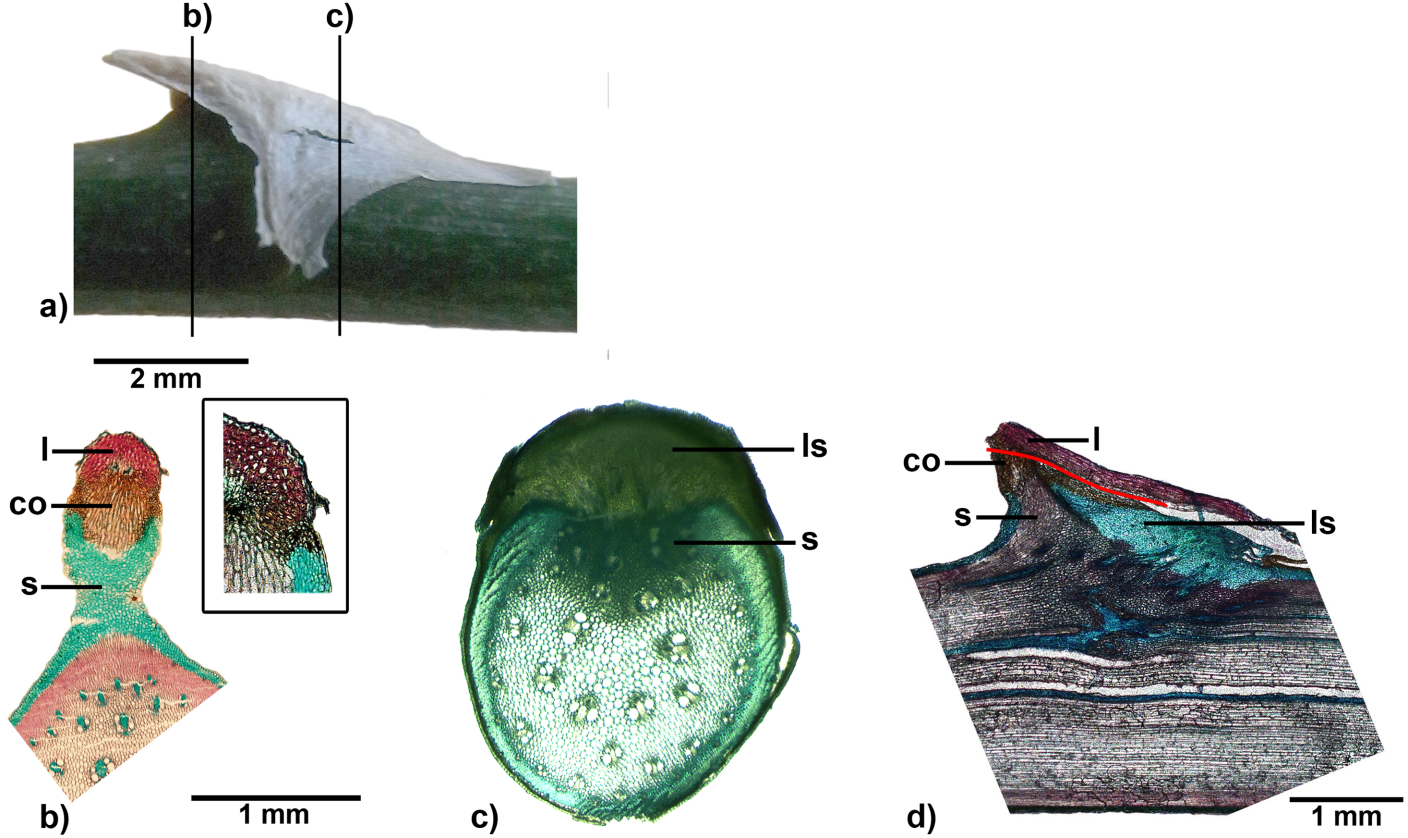
Spine of *Asparagus setaceus*. a) Side-view of a spine, lines indicate section planes of cross sections b) and c), b) and c) cross sections at different planes (from different specimens), b) stained with FCA, c) stained with toluidine blue (box: close up from a different cut), d) longitudinal section stained with toluidine blue. l: leaf tissue, s: stem tissue, ls: lateral shoot tissue, co: cork layer.

**Fig 11:**
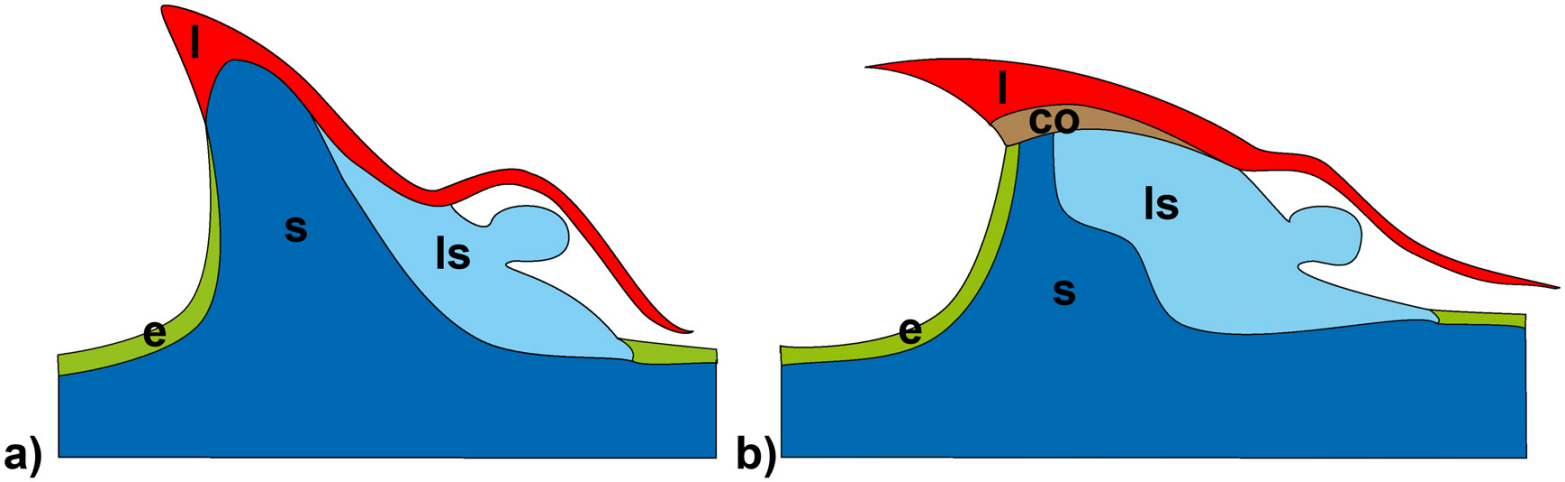
Schematic interpretation of the spine structures in *Asparagus falcatus* and *Asparagus setaceus*. The interpretation of the spine structures (a): *Asparagus falcatus*, b): *Asparagus setaceus*) is derived from longitudinal sections at different planes in different specimens. l: leaf tissue, s: stem tissue (including thick-walled lignified cells), ls: lateral shoot tissue, e: epidermis, co: cork layer.

In both *Asparagus falcatus* and *Asparagus setaceus* spines are composed of leaf tissue, stem tissue and tissue of the axillary bud / lateral shoot if already developed. (Figs 9–11). However, the proportions of the different tissues differ between the species with a higher contribution of leaf tissue and lateral shoot tissue in *A*. *setaceus*. Furthermore in *A*. *setaceus* a cork layer is formed between leaf and stem tissues of the spines, whereas no such cork layers were observed in spines of *A*. *falcatus*.

In both *Asparagus* species a gradual change of cell size and cell wall thickness in the spine itself was observed, with larger cells in the centre and smaller thick-walled cells towards the periphery (Figs 9 and 10).

**Fig 12:**
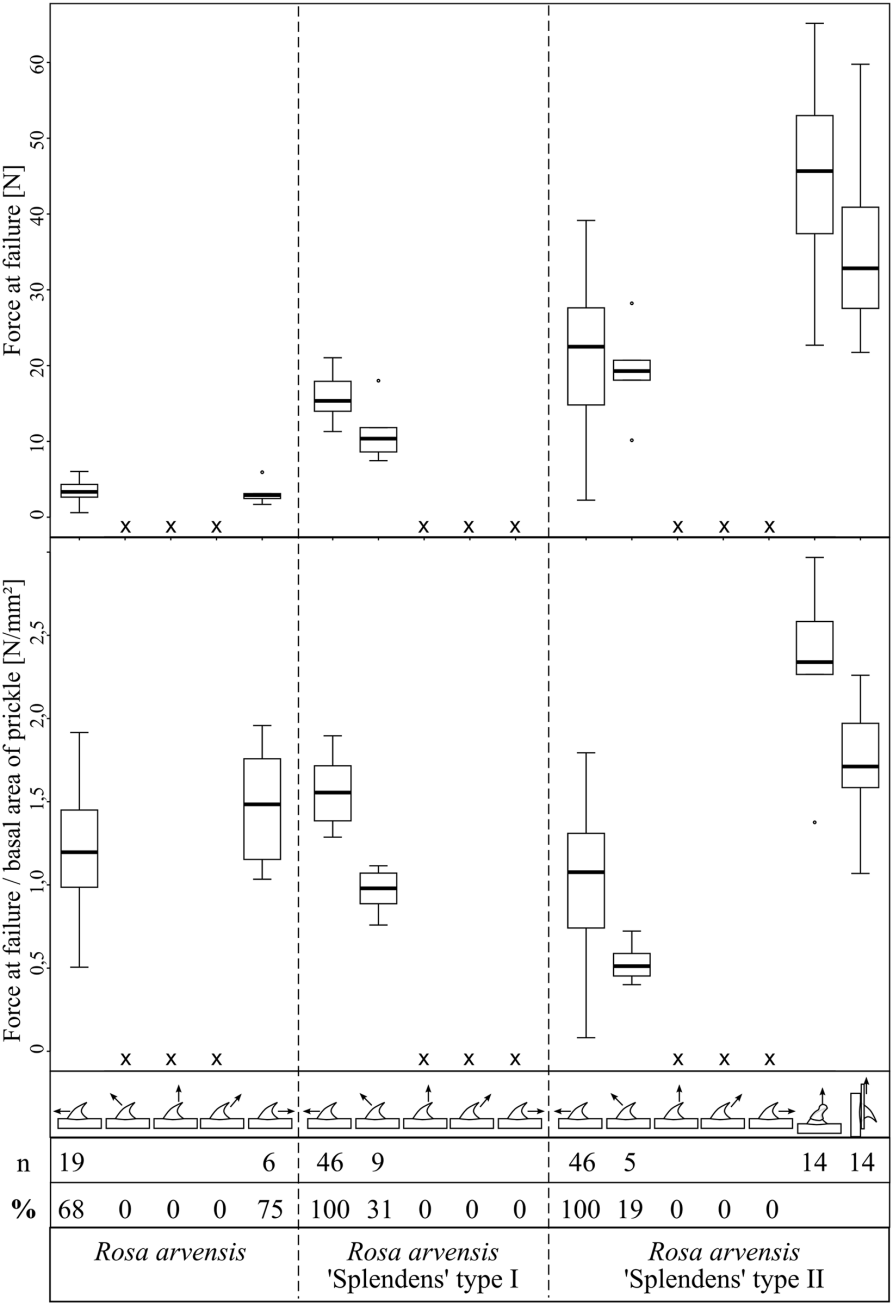
Maximum force and maximum force/basal area measured at failure of the tested rose prickles. In the illustrations of force direction the stem apex is situated on the left and the stem base on the right. x indicates that testing with a traction force in this direction was not possible due to slippage of the Kevlar loop. Numbers below indicate the number (n) and percentage (%) of samples having failed in the mechanical tests. In long axes type II of *Rosa arvensis* ‘Splendens‘ prickles were additionally tested in tension, with the prickle tested perpendicular to the stem, and in shear with the prickle tested parallel to the main axis of the stem (on the right).

The mechanical tests of the rose prickles (traction in different directions via a Kevlar loop, resulting in a combination of bending and shear stress) revealed differences in mechanical properties according to prickle type and also according to force orientation (Fig 12). Maximum forces at failure (F_max_) measured for the prickles of *Rosa arvensis* ‘Splendens‘ are significantly higher for the respective type of loading than maximum forces measured for prickles of the wild species *Rosa arvensis*. Within *Rosa arvensis* ‘Splendens‘ values of F_max_ are significantly higher in prickles of long axes type II than in prickles of long axes type I for respective types of loading (Kruskal-Wallis and Tukey’s-Test: p ≤ 0.001, Fig 12). However, when size differences of the prickles are accounted for by relating the values of maximum force measured at failure to the basal area of the prickles (F_max_/basal area) differences are not significant (comparing prickles of *Rosa arvensis* to prickles of *Rosa arvensis* ‘Splendens‘ type I). When the prickles of *Rosa arvensis* ‘Splendens’ type I are compared to the larger prickles of *Rosa arvensis* ‘Splendens’ type II the proportion of values is even reversed: Values of F_max_/basal area are significantly higher in prickles of *Rosa arvensis* ‘Splendens‘ type I than in prickles of *Rosa arvensis* ‘Splendens‘ type II (Kruskal-Wallis and Tukey’s-Test: p ≤ 0.001, Fig 12).

In *Rosa arvensis* failure of the prickles was only observed in experiments with an orientation of the traction force parallel to the stem (in both directions, towards the base and towards the apex of the stem), with failure of 68% and 75% of tested prickles in both directions respectively. The response of the prickles to these mechanical tests (failure by snapping off or non failure with slipping off of the Kevlar loop) correlates to differences in the angle formed between prickle and stem and in prickle size (Table 3). Prickles which did not fail because the Kevlar loop slipped off were smaller and more erect than prickles that failed by snapping off (Table 3). Traction with an angle of 45° (towards the base and towards the apex of the stem) or 90° resulted in the Kevlar loop slipping off in all tests (Fig 12). In both types of *Rosa arvensis* ‘Splendens‘ prickles, failure was observed when prickles were pulled with a traction parallel to the stem (100% of failure in tests with a traction force towards the apex of the stem) and also with a traction force at 45° (towards the apex of the stem, 31% in type I and 19% in type II). Prickles having snapped off in experiments with a traction force at 45° (towards the apex of the stem) are larger (significantly in type I) than those where the Kevlar loop simply slipped off (69% in type I and 81% in type II). In these tests no correlation between the response of the prickles to the mechanical tests (failure or non failure) and the angle formed between prickle and stem was found (Table 3). The fact that only the larger prickles have snapped off in tests with a traction at 45° results in smaller values of F_max_/basal area compared to values obtained with a traction force parallel to the stem (towards the apex of the stem), where 100% of the tested prickles finally failed (and not only the smallest). In all prickles submitted to traction applied via a Kevlar loop neither a bending of the prickle itself nor a deformation of the prickle base was observed (under a binocular) but in all such tests a deformation of the stem prior to failure of the prickle was recorded with a compression at one side of the prickle and an extension of stem tissue at the opposite side of the prickle.

**Table 3:** Geometry of rose prickles having failed and not failed.

*Rosa arvensis*	failed	not failed	p-value
angle [°]	83.3 (73.0, 91.1) n = 19	100.7 (95.0, 103.8) n = 9	***
height [mm]	3.8 (3.1, 4.2) n = 19	3.1 (2.6, 3.3) n = 9	*
basal area [mm²]	2.6 (1.9, 3.9) n = 19	2.0 (1.7, 2.3) n = 9	*
*Rosa arvensis* ‘Splendens‘ type I			
angle [°]	43.3 (38.6, 51.0) n = 9	46.7 (42.4, 55.2) n = 20	-
height [mm]	5.2 (4.6, 5.6) n = 9	4.5 (4.1, 4.7) n = 20	*
basal area [mm²]	9.9 (9.0, 13.0) n = 9	6.4 (5.7, 7.2) n = 20	***
*Rosa arvensis* ‘Splendens‘ type II			
angle [°]	55.0 (45.0, 59.0) n = 5	53.4 (50.8, 60.8) n = 21	-
height [mm]	8.4 (6.5, 9.8) n = 5	7.5 (86.8, 8.1) n = 21	-
basal area [mm²]	45.8 (25.0, 48.1) n = 5	25.8 (19.1, 36.3) n = 21	-

Prickles classified as failed have snapped off in mechanical tests. Prickles where the Kevlar loop slipped off are classified as not failed. For *Rosa arvensis* data obtained with traction parallel to the stem (force direction “1”) are pooled and for *Rosa arvensis* ‘Splendens‘ type I and II data obtained with traction at 45° (force direction “2”) are pooled. The specified values are medians and lower and upper quartiles (in brackets)

(***: p ≤0.001),

(*: p ≤0.05),

(-: P > 0.05, Mann-Whitney U-Test, Student’s t-test and Welch’s t-test).

**Fig 13:**
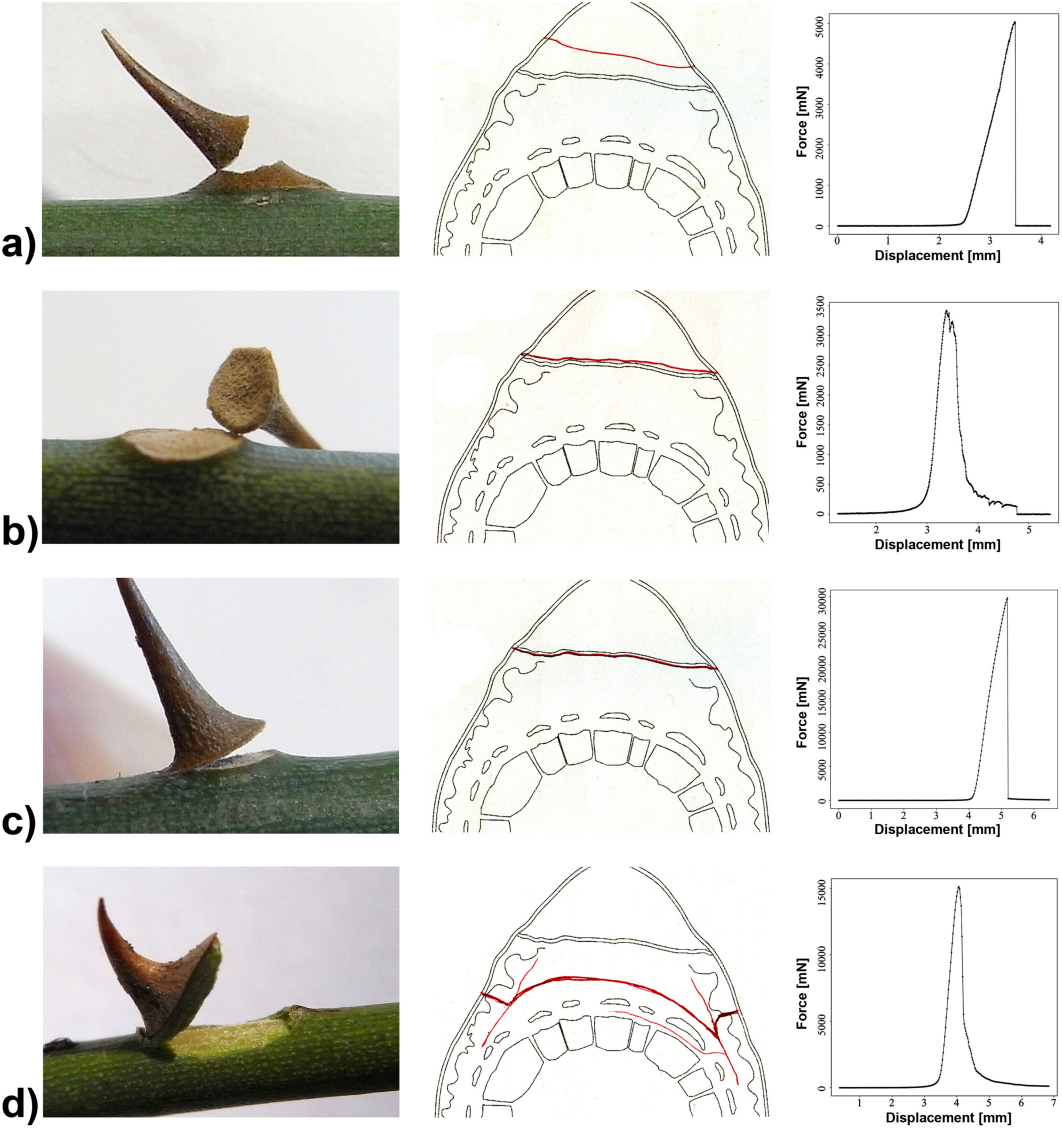
Modes of failure of rose prickles submitted to mechanical tests. Pictures, schematic drawings with typical crack location indicated by a red line and typical force-displacement-diagrams are shown. The initial displacements with almost zero force in the force-displacement diagrams represent the first testing phase when the Kevlar loop gets straightened before the force is exerted on the prickle. a) failure within the prickle (picture *Rosa arvensis*), b) failure just above the cork layer (picture *R*. *arvensis*), c) failure just beneath the cork layer (picture *R arvensis*) and d) failure within the cortical parenchyma of the stem (picture *Rosa arvensis* ‘Splendens‘ type II).

Patterns of failure were classified in four different modes: (1) failure within the prickle, (2) failure just above the cork layer, (3) failure just beneath the cork layer, and (4) failure within the stem tissue (Fig 13, Table 4). In the different prickle types different modes of failure prevailed. In prickles of *Rosa arvensis* 92% snapped with cracks within the prickle or above the cork layer, not running into the stem tissues and leaving the fractured surface of the stem covered with cork tissue (Table 4). In all snapped prickles of *Rosa arvensis* ‘Splendens‘ type I (tested with traction parallel to the stem or at 45°) cracks occurred within the cortical parenchyma and/or the phloem of the stem, thus causing substantial tissue damage in the stem (Fig 13, Table 4). In *Rosa arvensis* ‘Splendens‘ 38% of type II prickles snapped with cracks within the cortical parenchyma resulting in considerable damage to the stem tissue, whereas 62% had cracks just beneath the cork layer, thus leaving the stem tissue intact but without a marked cork layer covering the fracture surface (Fig 13, Table 4). These differences in the mode of failure do not correlate with differences in F_max_ or differences in bending moments. However, the mode of failure correlates to different geometrical parameters which all depend directly or indirectly on the prickle size (Table 5), whereby larger prickles predominantly snapped off above the cork layer. Consequently, all parameters related directly to prickle size (length of prickle base, basal area of prickles and lever arm) are significantly higher in prickles having snapped off above the cork layer whereas parameters divided by a size related parameter (F_max_/basal area, bending moment/basal area, deformation energy and deformation energy/basal area) are significantly smaller in prickles having snapped off above the cork layer (Table 5).

**Table 4:** Modes of failure in the different types of rose prickles.

Mode of failure	within prickle [%]	above cork layer [%]	beneath cork layer [%]	within stem tissue [%]
*Rosa arvensis* (n = 25)	56	36	8	-
*Rosa arvensis* ‘Splendens‘ type I (n = 58)	-	-	-	100
*Rosa arvensis* ‘Splendens‘ type II (n = 51)	-	-	62	38

**Table 5:** Geometrical and mechanical parameters related to the mode of failure in *Rosa arvensis* ‘Splendens‘ type II.

	failure beneath cork layer	failure within the cortical parenchyma	p-value
length of prickle base [mm]	10.9 ± 2.6 (n = 28)	9.0 ± 1.8 (n = 17)	**
basal area of prickles [mm²]	24.2 ± 10.6 (n = 28)	16.4 ± 4.6 (n = 17)	***
stem diameter [mm]	6.1 ± 1.2 (n = 28)	4.5 ± 0.2 (n = 17)	***
F_max_/basal area [N/mm²]	0.8 ± 0.3 (n = 28)	1.4 ± 0.2 (n = 17)	***
bending moment / basal area [N/mm]	3.3 ± 1.2 (n = 28)	4.1 ± 0.6 (n = 17)	**
deformation energy [N·mm]	3.7 ± 2.5 (n = 11)	9.2 ± 2.6 (n = 12)	***
deformation energy / basal area [N/mm]	0.2 ± 0.1 (n = 11)	0.5 ± 0.1 (n = 12)	***
lever arm [mm]	4.2 ± 1.2 (n = 28)	2.9 ± 0.6 (n = 17)	***

The specified values are mean values and standard deviations

(***: p ≤0.001),

(**: p ≤0.01, Student’s t-test and Welch’s t-test).

Mechanical tests in which *Rosa arvensis* ‘Splendens‘ type II prickles were submitted to tension perpendicularly to the stem or shear stress resulted in significantly higher values of F_max_ and F_max_/basal area at failure than tests in which the prickles were submitted to a combination of bending and shear stress by pulling them with a Kevlar loop in different directions (Fig 12). Both types of mechanical tests could only be performed when prickles were fastened and resulted in failure of the prickles with considerable damage within the cortical parenchyma of the stems.

**Fig 14:**
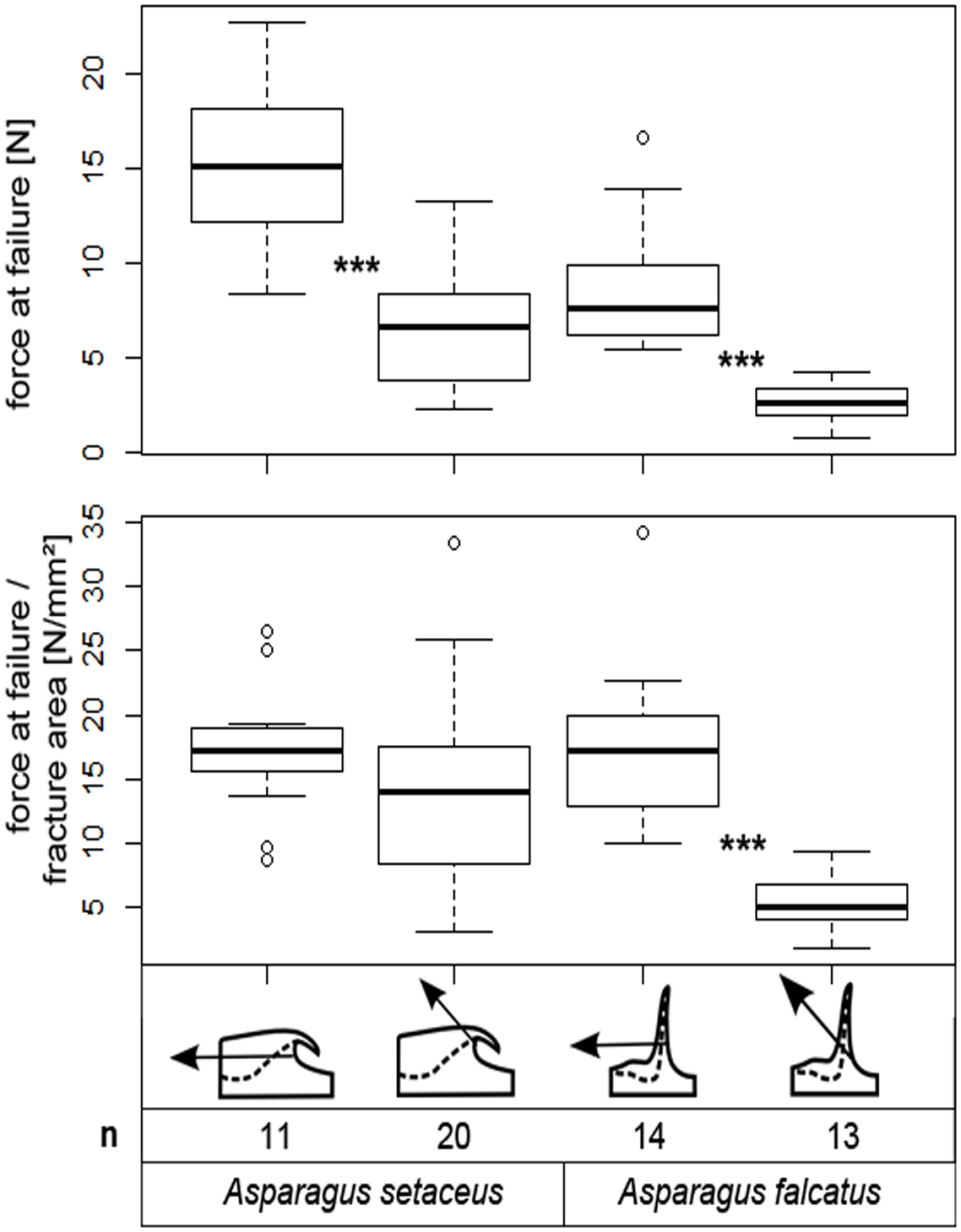
Maximum forces and maximum forces/basal area measured at failure in *Asparagus* spines. Maximum forces at failure were measured in *Asparagus falcatus* and *A*. *setaceus* with traction forces parallel to the stem and with an angle of 45° (toward the apex). Numbers below indicate the number (n) of samples (100% in all tests) having failed. (***: p ≤0.001, Student’s t-test and Mann-Whitney U-test).

In both *Asparagus setaceus* and *Asparagus falcatus* mechanical tests by pulling the spines with a Kevlar loop resulted in 100% failure of the spines when the traction force was applied parallel to the stem and with an angle of 45° (towards the apex, with significantly higher values of F_max_ obtained with a traction force parallel to the stem in both species (p < 0.001, Fig 14) and significantly higher values of F_max_/fraction area obtained with a traction force parallel to the stem in *A*. *falcatus* (p < 0.001, Fig 14).

Furthermore, comparison of spines composed of a modified leaf and tissue of an axillary bud with spines composed of a modified leaf and tissue of an already developed lateral shoot revealed no significant differences of F_max_ and F_max_/fraction area at failure in both species.

**Fig 15:**
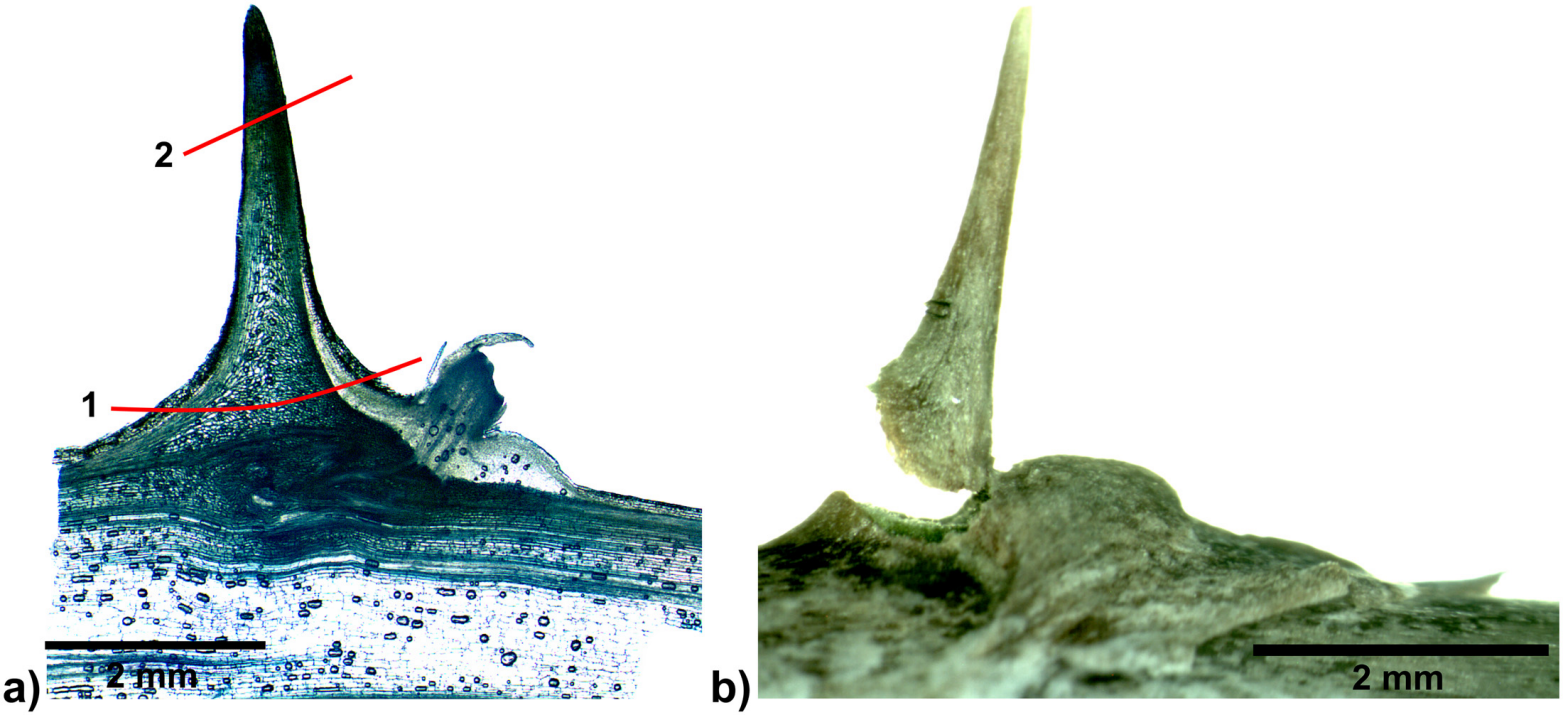
Modes of failure in spines of *Asparagus falcatus*. a) longitudinal cut, the red lines indicate failure at the base (1), and failure at the tip (2), respectively, b) typical appearance of a spine having snapped off at the base.

**Fig 16:**
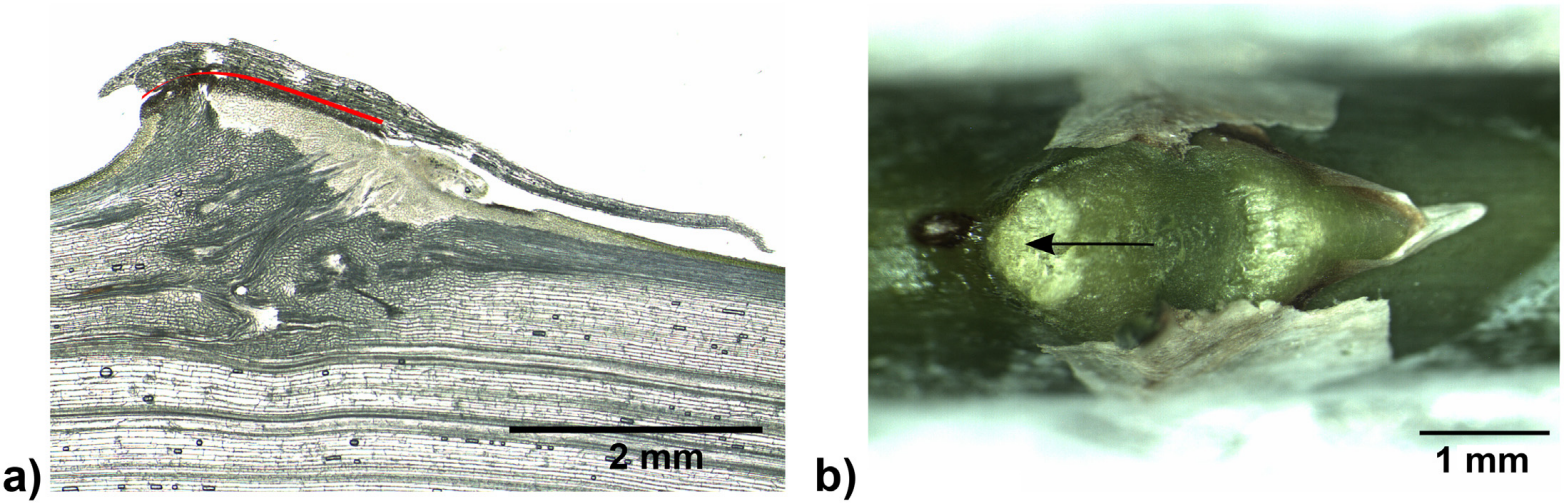
Typical mode of failure in spines of *Asparagus setaceus*. a) longitudinal cut, red line indicating failure along the cork layer (failure at the tip not shown), b) typical appearance of the fracture area left by failure along the cork layer. The arrow indicates the small spot of damaged stem tissue being characteristically for this mode of failure.

In the tested *Asparagus* species three different modes of failure of the spines were identified: (1) failure of the whole spine (predominant mode of failure in *A*. *falcatus*), (2) failure along the cork layer (predominant mode of failure in *A*. *setaceus* when spines were pulled with a traction force parallel to the stem), and (3) failure at the tip (observed for a small proportion of spines in *A*. *falcatus* and predominant in *A*. *setaceus* when spines were pulled with a traction force of 45°) (Figs 15 and 16, Table 6). Although no cork layers were observed in *A*. *falcatus* spines failed with a similar appearance and position of the fraction area in all tests (Fig 15). Likewise, in *A*. *setaceus* spines having been “peeled off” along the cork layer left a similar fracture area with a small spot of damaged stem tissue (Fig 16). In both *Asparagus* species the proportions of the different modes of failure varied according to the direction of the applied traction force (Table 6).

**Table 6:** Modes of failure of the spines observed in the tested Asparagus spines.

Mode of failure	failure of the whole spine [%]	failure along cork layer [%]	failure at the tip [%]
traction force parallel to the stem			
*Asparagus falcatus* (n = 13)	100	-	-
*Asparagus setaceus* (n = 11)	-	64	36
traction force with an angle of 45°			
*Asparagus falcatus* (n = 13)	77	-	23
*Asparagus setaceus* (n = 20)	-	5	95

## Discussion

Our results as to general morphology and anatomy of the prickles of *Rosa arvensis* and the cultivar *Rosa arvensis* ‘*Splendens*‘ are consistent with observations stated for prickles of other rose species (e.g. by [11]), indicating that the internal structure of rose prickles is not subject to marked interspecific variation. In contrast, the compared prickles differ significantly in size, geometry, orientation and mechanical properties not only between the wild species and its cultivar but also between different types of long axes within one individual of the cultivar. It remains to be investigated to what extent such differences are correlated to different attachment properties on various surfaces and therefore can be interpreted as adaptation to different ecological niches of different specimens or even parts of one specimen in climbing *Rosa* species. However, it has to be taken into account that the cultivar *Rosa arvensis* ‘*Splendens*‘ has been bred and selected for use in horticulture. This may also account for the finding, that its prickles typically cause damage of the stem tissues when snapping off, which can be hypothesized to be a disadvantage under natural conditions. Stem damage is not observed in the wild species, at least not when the prickles are submitted to mechanical stresses that are likely to occur under natural conditions by pulling them at different angles and thus generating a combination of bending and shear stresses (which also happens when prickles get caught in irregularities of the surfaces of supporting structures). However, it has to be taken into account that under natural conditions stems attached to a support by their hooks are able to “escape” overcritical mechanical loads by elastic deformation causing an unlocking of the hooks, whereas in the experimental setup stems were fastened. Submission of fastened prickles to pure shear stress or pure tensional stress resulted in significantly higher values of F_max_ and considerable stem tissue damage but can be considered as a form of mechanical stress only rarely occurring under natural conditions.

The spines of *Asparagus setaceus* and *Asparagus falcatus* also differ significantly in size, geometry, orientation and mechanical properties. As reported by [6, 7] they can be considered as modified leaf structures. However, the analysis of the anatomy of spines in *A*. *setaceus* and *A*. *falcatus* reveals that in these species the spines are also composed partly of stem tissues and tissues of the axillary bud or lateral shoot, respectively. The latter may possibly act as counter bearings when the spines are submitted to mechanical stresses (leading to compression at the adaxial sides of the spines where the axillary buds are located, because all spines are orientated downwards). In comparison to mean values of maximum force at failure reported for leaf hooks of *Galium aparine*: (< 0,03 N, [3]) or for acanthophylls of *Desmoncus* species (up to 102 N, [12]) the values measured for rose prickles and *Asparagus* spines are in an average force range.

The hook structures in *Rosa arvensis* and *Rosa arvensis* ‘Splendens‘ do not vary in shape and orientation along the studied long axes. This is consistent with the fact, that the tested *Rosa* species and its cultivar are woody plants developing stems of several meters length and that the observed long axes are lateral branches that may contribute to the attachment of the whole plant to its supporting structures throughout their entire lengths. In hooks serving as defence against herbivory a preferential orientation is not necessary. Therefore the observed reorientation of the prickles into structures bent towards the base of the stems during the first days of development may present an adaptation essentially improving their functioning as attachment devices. However, a simultaneous function as defence against herbivory can be assumed for the prickles which densely cover the stems (and leaf petioles) of *Rosa* species. This is consistent with the observation, that the prickles also develop in basal segments of main stems, which are not involved in the attachment of the plant to surrounding supporting structures, but may be exposed to herbivory. On the contrary, the basal stem segments of the herbaceous *Asparagus* species which are also not involved in the attachment of the plants to their supporting structures do not bear fully developed spines. These stems, as typical in semi-self-supporting “leaning plants” [1, 2], are mechanically stable in basal parts (and in early ontogenetic stages) and start to “lean on” structures of the surrounding vegetation only with the upper parts of their plant body (in later ontogenetic stages). With regard to the attachment function the development of fully grown spines can therefore be “economized” in lower parts of the *Asparagus* stems. A major role in defence against herbivory seems less apparent for the spines of the studied *Asparagus* species, as in comparison to the *Rosa* prickles they are dispersed only scarcely along the stems, exposing longer stem segments without defence.

Although the studied species belong to only very distantly related Angiosperm lineages (Monocots and Dicots), and although their hook structures are of different structural origins (prickles developing from epidermal tissue versus spines developing from leaf and stem tissue) their hook structures have several traits in common. (1) Their inner structure displays a gradual change of cell size and cell wall thickness, with larger cells in the centre and smaller thick-walled cells at the periphery of the hooks, (2) They occur in a diversity of shape and geometry within one individual (although this is less apparent in *Asparagus setaceus*), (3) single hooks fail when submitted to moderate mechanical stresses (F_max_/basal area < 35 N/mm²); and (4) failure of the hooks does not cause severe stem damage (at least in the tested wild species and when submitted to stresses that are comparable to stresses occurring under natural conditions). It is likely that these characteristics represent selective advantages in hooks functioning as attachment devices in general.

The development of gradients in cell size and cell wall thickness and consequently in mechanical properties from the centre of the prickles and spines (where mechanical stresses are low) towards the periphery (where mechanical stresses are highest) has been observed also in many other plant organs such as culms of different Poaceae or the stems of Arecaceae and shown to be related to differences in mechanical properties across the stems [13–17]. It can be hypothesized that in the studied *Rosa* prickles and *Asparagus* spines the observed gradients in anatomical parameters may also account for an improvement of the mechanical stability of these structures. A detailed analysis of this hypothesis will be subject of further studies including finite element simulation of prickles and spines.

Differences in geometry and size correlate with differences in the response to mechanical stresses (these correlating to different force directions and different modes of failure). It can be assumed that under natural conditions even within one plant a diversity of different mechanical stresses (with different force directions, resulting in bending, tension, and shear stresses and/or combinations of these) act on different hook structures. It is also likely, that under natural conditions the tips of the hooks engage directly with rough surfaces, resulting in mechanical loading with a higher bending moment than tested here (with the Kevlar loop positioned at the vertex point). Under these conditions the development of prickles or spines differing in size and geometry (and responding differently to mechanical stresses) may represent an advantage when individual hook structures are attached to different surfaces, at various angles and submitted to different mechanical stresses, thus securing the attachment of the whole plant even when single attachment structures snap off. It can be assumed that in semi-self-supporting plants as the studied *Rosa* and *Asparagus* species the loss of single hook structures does not weaken the attachment of the whole stem (or plant) in a critical manner, whereas injuries of stem tissues may represent a greater threat. In this respect the hook structures of semi-self-supporting plants differ fundamentally from attachment devices of climbing plants which establish a much closer attachment to the host plants which is necessary as these climbers entirely have lost mechanical (self-)stability of their stems in older ontogenetic stages. Coiling tendrils, adventitious roots with root hairs or adhesive pads secure a much closer attachment to the host plants and adhere much firmer to their supporting structures [1, 18–22]. In these plants typically flexible structures (e.g. tendril coils, roots and root hairs or deformable tissues) are interposed between the direct attachment devices and the supporting structure, which act as a combination of spring and dashpot, and help for reducing the mechanical stresses acting on the attachment devices. Here failure predominantly occurs in such interposed structures [1, 20–23]. On the contrary, prickles and spines are designed as stiff structures that can get caught efficiently in surface irregularities of the supporting structures. Due to the stiffness of the hook structures and their position on the stems, movements of the stems provoke mechanical stresses acting directly on the prickles or spines and the closely connected stem tissues with no damping elements in between. In this case damages of the stem tissue are prevented by a failure of the hook structures at relatively low to moderate mechanical stresses. This strategy implies a (relative) loose attachment to the surrounding plants and a huge number of attachment elements of which several can fail without markedly reducing the overall attachment. This kind of attachment structures found in the studied *Rosa* and *Asparagus* species is typical for many semi-self-supporting plants [1, 2, 4].

## Supporting Information

S1 File Raw data *Rosa arvensis* and *Rosa arvensis* ‘Splendens’. (XLS)

S2 File Raw data *Asparagus setaceus* and *Asparagus falcatus*. (XLSX)
